# Potential of Minimally Invasive Tissue Sampling for Attributing Specific Causes of Childhood Deaths in South Africa: A Pilot, Epidemiological Study

**DOI:** 10.1093/cid/ciz550

**Published:** 2019-10-09

**Authors:** Richard Chawana, Vicky Baillie, Alane Izu, Fatima Solomon, Quique Bassat, Dianna M Blau, Robert F Breiman, Martin Hale, Eric R Houpt, Sanjay G Lala, Roosecelis B Martines, Azwifarwi Mathunjwa, Susan Nzenze, Jayani Pathirana, Karen L Petersen, Pratima L Raghunathan, Jana M Ritter, Jeannette Wadula, Sherif R Zaki, Shabir A Madhi

**Affiliations:** 1 Medical Research Council, Respiratory and Meningeal Pathogens Research Unit, University of the Witwatersrand, Faculty of Health Sciences, Johannesburg, South Africa; 2 Department of Science and Technology/National Research Foundation, Vaccine Preventable Diseases, University of the Witwatersrand, Faculty of Health Sciences, Johannesburg, South Africa; 3 ISGlobal, Hospital Clínic, Universitat de Barcelona, Barcelona, Spain; 4 Centro de Investigação em Saúde de Manhiça (CISM), Maputo, Mozambique; 5 Catalan Institution for Research and Advanced Studies (ICREA), Barcelona, Spain; 6 Pediatric Infectious Diseases Unit, Pediatrics Department, Hospital Sant Joan de Déu, Universitat de Barcelona, Barcelona, Spain; 7 Consorcio de Investigacion Biomedica en Red de Epidemiologia y Salud, Spain; 8 Center for Global Health, Centers for Disease Control and Prevention, Atlanta, Georgia, USA; 9 Emory Global Health Institute, Emory University, Atlanta, Georgia, USA; 10 National Health Laboratory Service, Department of Anatomical Pathology, School of Pathology, Faculty of Health Sciences, University of the Witwatersrand, Johannesburg, South Africa; 11 Division of Infectious Diseases and International Health, University of Virginia, Charlottesville, USA; 12 Department of Paediatrics, and University of the Witwatersrand, Johannesburg, South Africa; 13 Perinatal HIV Research Unit, Chris Hani Baragwanath Academic Hospital, University of the Witwatersrand, Johannesburg, South Africa; 14 Infectious Diseases Pathology Branch, Division of High-Consequence Pathogens and Pathology, National Center for Emerging and Zoonotic Infectious Diseases, Centers for Disease Control and Prevention, Atlanta, Georgia, USA; 15 National Health Laboratory Service, Department of Microbiology and Infectious Diseases, School of Pathology, Faculty of Health Sciences, University of the Witwatersrand, Johannesburg, South Africa

**Keywords:** child mortality, minimally invasive tissue sampling, pneumonia, diarrhea, South Africa

## Abstract

**Background:**

Current estimates for causes of childhood deaths are mainly premised on modeling of vital registration and limited verbal autopsy data and generally only characterize the underlying cause of death (CoD). We investigated the potential of minimally invasive tissue sampling (MITS) for ascertaining the underlying and immediate CoD in children 1 month to 14 years of age.

**Methods:**

MITS included postmortem tissue biopsies of brain, liver, and lung for histopathology examination; microbial culture of blood, cerebrospinal fluid (CSF), liver, and lung samples; and molecular microbial testing on blood, CSF, lung, and rectal swabs. Each case was individually adjudicated for underlying, antecedent, and immediate CoD by an international multidisciplinary team of medical experts and coded using the *International Classification of Diseases*, *Tenth Revision* (*ICD-10*).

**Results:**

An underlying CoD was determined for 99% of 127 cases, leading causes being congenital malformations (18.9%), complications of prematurity (14.2%), human immunodeficiency virus/AIDS (12.6%), diarrheal disease (8.7%), acute respiratory infections (7.9%), injuries (7.9%), and malignancies (7.1%). The main immediate CoD was pneumonia, sepsis, and diarrhea in 33.9%, 19.7%, and 10.2% of cases, respectively. Infection-related deaths were either an underlying or immediate CoD in 78.0% of cases. Community-acquired pneumonia deaths (n = 32) were attributed to respiratory syncytial virus (21.9%), *Pneumocystis jirovecii* (18.8%), cytomegalovirus (15.6%), *Klebsiella pneumoniae* (15.6%), and *Streptococcus pneumoniae* (12.5%). Seventy-one percent of 24 sepsis deaths were hospital-acquired, mainly due to *Acinetobacter baumannii* (47.1%) and *K. pneumoniae* (35.3%). Sixty-two percent of cases were malnourished.

**Conclusions:**

MITS, coupled with antemortem clinical information, provides detailed insight into causes of childhood deaths that could be informative for prioritization of strategies aimed at reducing under-5 mortality.

## Teaser

Using minimally invasive tissue sampling in children 1 month to 14 years of age, cause of death (CoD) was determined in 99% of cases. The leading underlying CoDs were congenital malformations, complications of prematurity, HIV/AIDS, diarrheal disease, and acute respiratory infections. The main immediate CoDs were pneumonia, sepsis, and diarrhea. Infection-related deaths were either an underlying or immediate CoD in 78% of cases.

Global mortality rates in children aged 28 days to 1 year and 1–5 years have declined between 2000 and 2015 at an annualized rate of 2.8% and 3.5%, respectively, including 4.4% and 5.4% in respective age groups in sub-Saharan Africa [1, 2]. Current estimates on causes of under-5 childhood deaths are largely premised on modeling using vital registration data coupled with limited verbal autopsy data (approximately 1 per 850 deaths) [3], exposure prevalence, and risk-attribution factors [3, 4]. Furthermore, the estimates on causes of childhood deaths are mainly focused on the underlying medical condition that likely led to the sequence of events resulting in death. This could undermine recognition of more immediate medical events that resulted in the death, which might otherwise be preventable or for which interventions can be developed. The current dependency on verbal autopsy data, a nonspecific method for cause of death (CoD) attribution, argues for more objective and granular understanding of specific immediate causes of death in low- and middle-income countries (LMICs).

Complete diagnostic autopsy is the “gold standard” for ascertaining CoD, but is rarely undertaken in LMICs due to limited resources, cost constraints, and cultural and religious barriers on acceptability [5, 6]. Minimally invasive tissue sampling (MITS), also known as minimally invasive autopsy, involves postmortem collection of fluid and solid tissue samples using biopsy needles. Community engagement studies have suggested that MITS would be theoretically culturally acceptable in African and South Asian countries [6]. Furthermore, a validation study undertaken in Mozambique demonstrated moderate concordance (κ = 0.75) in CoD attribution between complete diagnostic autopsy and MITS in children >1 month of age [7].

We aimed to evaluate the acceptability and utility of MITS, as proof of concept, in ascertaining the causal pathway of death in children >1 month of age in an LMIC setting (Soweto, South Africa).

## METHODS

### Study Site and Population

The study was conducted from 29 June 2015 to 1 August 2016 at Chris Hani Baragwanath Academic Hospital (CHBAH). Further details of the study site, including healthcare setting and human immunodeficiency virus (HIV) prevalence, are given in the companion manuscript on neonatal deaths, also published in this supplement [8]. Additionally, in South Africa, the Expanded Programme on Immunization includes pneumococcal conjugate vaccine (PCV) and rotavirus vaccine that have been introduced since 2009, in addition to 8 other routinely administered childhood vaccines [9].

### Study Design and Procedures

In this prospective, observational study, deaths of children <14 years of age occurring in the medical and emergency department of CHBAH, and those certified as dead upon arrival at the hospital, were identified through daily screening of death registries, excluding from 18 December 2015 to 3 January 2016 (vacation period). We initiated the study by initially only screening and enrolling deaths occurring in the general medical wards. The surveillance and enrollment of cases was expanded to include deaths occurring in the surgical burn unit (31 August 2015), hematology/oncology ward (16 September 2015), surgical wards (30 September 2015), and the nursery ward for care of stable very low-birth-weight infants (kangaroo-care facility; 7 October 2015). Details on the counseling and consenting processes are as detailed elsewhere in the companion paper on neonatal deaths [8].

We aimed to undertake MITS within 24 hours of death and excluded those cases in which the procedure could not be performed within 72 hours. Corpses were kept in the hospital mortuary at 4°C until retrieval for burial.

### Minimally Invasive Tissue Sampling

Trained study staff (medical doctor or professional nurse assisted by research assistants) undertook MITS as detailed elsewhere in this supplement [8]. The testing algorithm for the collected samples was similar to that done in neonatal deaths, with the exception that molecular testing of blood samples using the FastTrack Diagnostics (FTD; Sliema, Malta) neonatal sepsis kit was not undertaken in the age group 1 month to 14 years; and only the FTD Respiratory-33 panel kit was used on lung samples. Methods used for tissue histology processing are also described elsewhere in this supplement [8].

### Determination of Cause of Death

The CoD attribution was based on consensus of an international panel convened in South Africa from 26 March to 5 April 2017 composed of pathologists, pediatricians, epidemiologists, microbiologists, obstetricians, infectious disease specialists, and international coding and certification experts (the Determination of Cause of Death [DeCoDe] panel group is listed in the Acknowledgements). The panel, chaired by either Chris Wilson or Scott Dowell, deliberated on CoD attribution using the *International Classification of Diseases, Tenth Revision* (*ICD-10*) [10]. This included attributing a single underlying medical condition that likely initiated the sequence of events culminating in death, and the most proximal event (ie, immediate cause) of death, if applicable. Also evaluated were any antecedent conditions in the casual pathway leading to death, and medical conditions considered to have a contributory role, but not directly involved in the causal pathway. Details on the working methods of the DeCoDe panel are described in the companion manuscript on causes of neonatal deaths [8]. The DeCoDe panel allowed for inclusion of two “immediate” CoD, where concurrent diseases could not be prioritized as the main condition that caused the death; for example, histologically confirmed *Pneumocytis jirovecii* and respiratory syncytial virus (RSV) pneumonitis being concurrently present (and attributed as the immediate CoD) in a child with underlying HIV infection (attributed as the underlying CoD).

The CoD was generally based on consensus, in the absence of which a majority viewpoint was used, and recorded according to World Health Organization (WHO) guidelines for death certification. In addition, CoD was stratified into first-level United Nations Inter-agency Group on Child Mortality Estimation (UN-IGME) categories [11]: group I (an aggregation of communicable, maternal, perinatal, and nutritional conditions), group II (noncommunicable diseases), and group III (injuries).

### Statistical Analysis

We stratified analyses to infants (1–11 months of age), children (12–59 months of age), and older children (60 months to 14 years of age). Descriptive statistics were calculated providing medians with interquartile ranges for continuous variables, and proportions for categorical variables. For select variables, differences between the infants, children, and older children were tested using Kruskal-Wallis or Fisher exact test. Weight-for-age was calculated in Stata software using the UK WHO Preterm Growth Charts (version UKWHOpreterm) and UK WHO Term Growth Charts (version UKWHOterm) for the preterm babies and children aged 0–20 years, respectively. Statistical analysis was done using Stata software version 15 (StataCorp, College Station, Texas).

### Ethical Considerations

The University of the Witwatersrand Human Research Ethics Committee (number 150215) and the CHBAH Protocol Review Committee approved the study. Parents/legal guardians provided consent prior to any MITS study procedure.

## RESULTS

**Figure 1.**
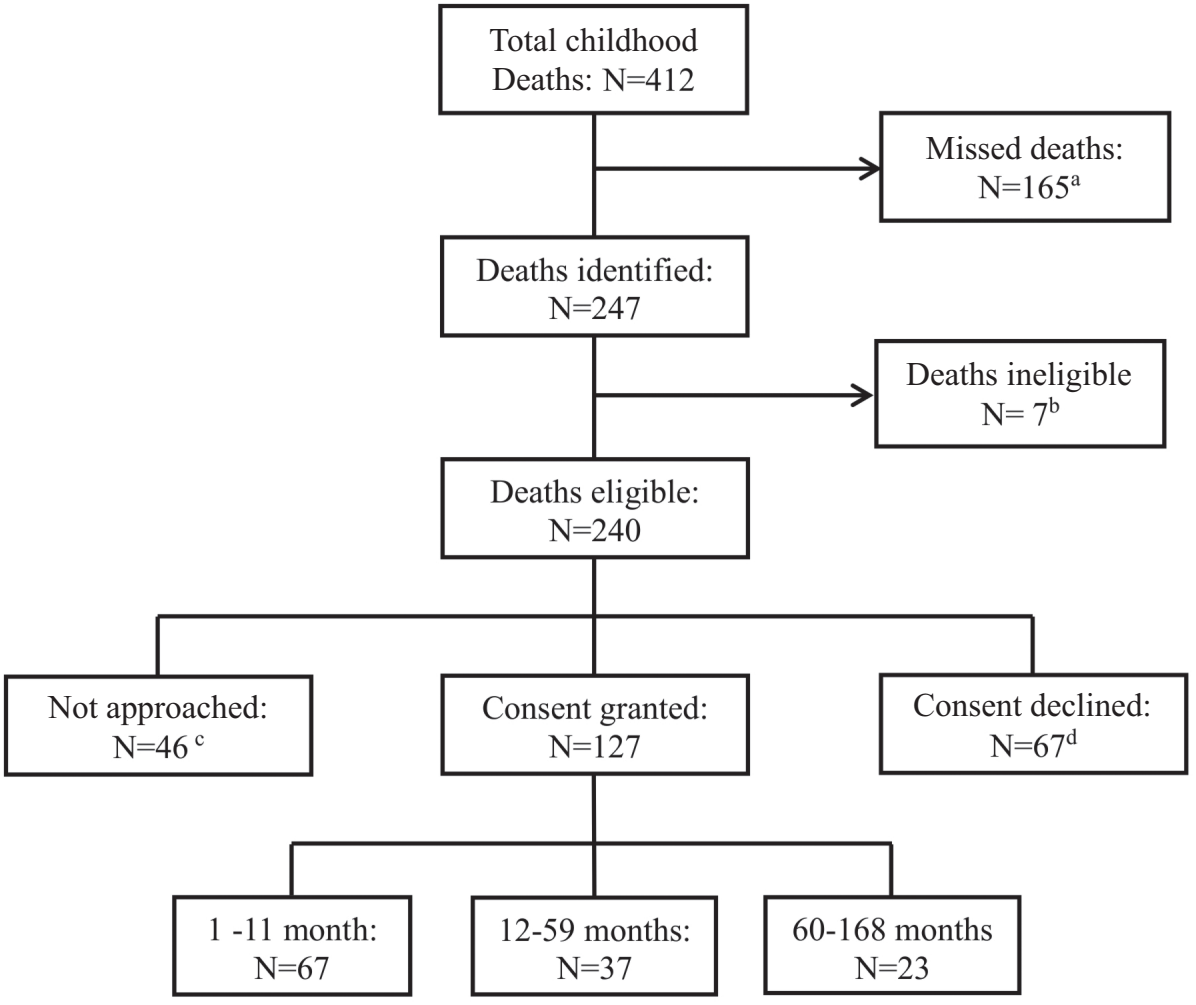
Flow diagram showing death notifications at the Chris Hani Baragwanath Academic Hospital of children aged 1 month to 14 years, and screening and enrollment of cases into the minimally invasive tissue sampling study. ^a^Deaths were missed because the initial surveillance was limited to the pediatric medical wards, high care unit, and casualty (emergency ward). The surveillance was later extended to the burns unit (on 31 August 2015), hematology/oncology ward (16 September 2015), surgical ward (30 September 2015), and the neonatal kangaroo-care nursing ward (7 October 2015) to capture all childhood deaths at the facility. ^b^Ineligible cases included a medico-legal death (n = 1), a case from outside Soweto (n = 1), those with no legal guardian (n = 2), and cases where both parents were minors (n = 3). ^c^We enrolled cases on all days, excluding weekends and public holidays, from 19 December 2015 to 2 January 2016, during which time 24 cases were not approached for consenting. Additionally, 4 children were buried at the time of contact and we could not contact the parents of 18 cases within 72 hours of death. ^d^Most parents did not provide a reason for declining study participation (n = 51 [76.1%]). Reasons cited for nonparticipation included cultural reasons (n = 9 [13.4%]), parental belief that they were aware of the cause of death (n = 3 [4.5%]), and parental feeling of the child already having suffered (n = 4 [6.0%]).

There were 412 deaths among children aged 1 month to 14 years at CHBAH during the study period. Sixty percent (n = 247) of the deaths were screened for study participation, while 165 (40%) cases were not screened mainly due to the timing of phasing in of enrollment in the nonmedical wards (Figure 1). Seven of the 247 (2.8%) screened cases were ineligible, and in 19.2% cases the parents could not be contacted within 72 hours of death. Of the remaining 194 screened cases, 127 (65.5%) consented to study participation, including 14 of 127 (11.0%) who were certified dead upon hospital arrival.

The median age of enrolled cases was 11 months; 52.8% were infants, 29.1% children, and 18.1% older children. The median time from hospital admission to death was 3.0 days. MITS sampling was done at a median of 20.2 (11.3–27.6) hours after death. Overall, 36.4% of cases were born to women living with HIV; however, only 12.8% of cases were confirmed to be HIV infected on postmortem HIV polymerase chain reaction testing. Overall, 62.4% of cases, including 71.7% of infants, were malnourished. Sixteen percent of cases had underlying congenital abnormalities. Approximately 20% of the cases had been admitted for mechanical ventilation support in the intensive care unit (Table 1).

**Table 1.** Demographic Features of Childhood Deaths in Which Minimally Invasive Tissue Sampling Was Undertaken

Features	Total	Infants (1–11 mo)	Children (12–59 mo)	Older Children (≥60 mo)
	(N = 127)	(n = 67)	(n = 37)	(n = 23)
Median age at death, mo (IQR)	11.0 (2.6–26.9)	2.7 (1.8–5.7)	19.2 (13.7–26.5)	97.9 (77.1–105.2)
Male sex, No. (%)	64 (50.4)	31 (46.3)	22 (59.5)	11 (47.8)
HIV exposed^a,b^, no./No (%)	40/110 (36.4)	26/64 (40.6)	9/30 (30.0)	5/16 (31.3)
HIV infected^c^, no./No (%)	16/125 (12.8)	9 (13.4)	3 (8.1)	4/21 (19.0)
Median weight, kg at admission (IQR)	5.1 (2.5–9.3)	2.8 (1.4–4.5)	8.7 (6–10)	17.3 (14.1–22.6)
Weight for age^d^*z* score < –2, no./No (%)	53/85 (62.4)	33/46 (71.7)	14/27 (51.9)	6/12 (50.0)
Median No. of days between admission and death (IQR)	3 (0–28)	7 (0–40)	1 (0–4)	5 (1–12)
Median time between death and MITS done, h	20.2 (11.3–27.6)	23.0 (14.3–28.2)	18.0 (7.0–24.1)	16.6 (11.7–28.0)
Congenital abnormalities present^e^, No. (%)	20 (15.7)	13 (19.4)	5 (13.5)	2 (8.7)
Mechanical ventilation support during hospitalization^f^, no./No (%)	21/103 (20.4)	19/53 (35.9)	2/31 (6.5)	0/19 (0.0)
Days spent in ICU^g^ (IQR)	32 (11–54)	32 (11–62)	19 (4–34)	Not applicable
Time to death between ICU discharge and death, d	0 (0–1)	0 (0–1)	9 (0–18)	Not applicable

Abbreviations: HIV, human immunodeficiency virus; ICU, intensive care unit; IQR, interquartile range; MITS, minimally invasive tissue sampling.

^a^HIV exposed includes children born to HIV-infected mothers or children who were HIV polymerase chain reaction negative but HIV enzyme-linked immunosorbent assay positive.

^b^HIV exposure could not be ascertained in 17 of the 127 cases.

^c^No HIV result in 2 cases. One case was born to an HIV-uninfected woman and the HIV exposure status of the other case was unknown.

^d^United Kingdom (UK) World Health Organization (WHO) preterm and term growth charts were used to calculate *z* scores for the preterm babies and children 0–20 years, respectively.

^e^The congenital malformations were identified from the case notes or during the MITS procedure. These included 6 cases of hydrocephalus (3 in infants, 1 in children, and 2 in older children), 1 of which also had other additional malformations of Hirchsprung disease (1 infant). Three cases had Down syndrome (1 infant and 2 children); 2 cases had unspecified dysmorphic features including 1 with cleft palate (both infants); and 1 case each had exomphalos with jejunal atresia (infant), anorectal malformations (child), biliary atresia (child), duodenal atresia with patent ductus arteriosus (infant), jejunal atresia (infant), gastroschisis (infant), choanal atresia with patent ductus arteriosus (infant), spina bifida (child), and transposition of great vessels (child).

^f^There were no records in 24 cases to identify whether the child received mechanical ventilation. Mechanical ventilation modalities included 8 cases that received continuous positive airway pressure (7 infants and 1 child), 7 positive end pressure (6 infants and 1 child), 4 unspecified ventilation type (4 infants), and 1 each of high-frequency oscillatory ventilation (1 infant) and intermittent positive-pressure ventilation (1 infant).

^g^Median duration of stay for those children who were admitted to ICU.

### MITS Tissue Adequacy

For solid tissues, most tissue biopsy samples were graded as adequate: 71.6% (n = 91) for left lung, 77.2% (n = 98) for right lung, 81.9% (n = 104) for brain, and 84.2% (n = 107) for liver; or as suboptimal: 8.7% (n = 11) for liver, 11% (n = 14) for brain, 15.7% (n = 20) for right lung, and 19.7% (n = 25) for left lung. No target organ tissue was obtained in 5.5% (n = 7) of liver, 7.1% (n = 9) of right lung and brain, and 8.7% (n = 11) of left lung samples.

### Cause of Death Attribution

**Figure 2.**
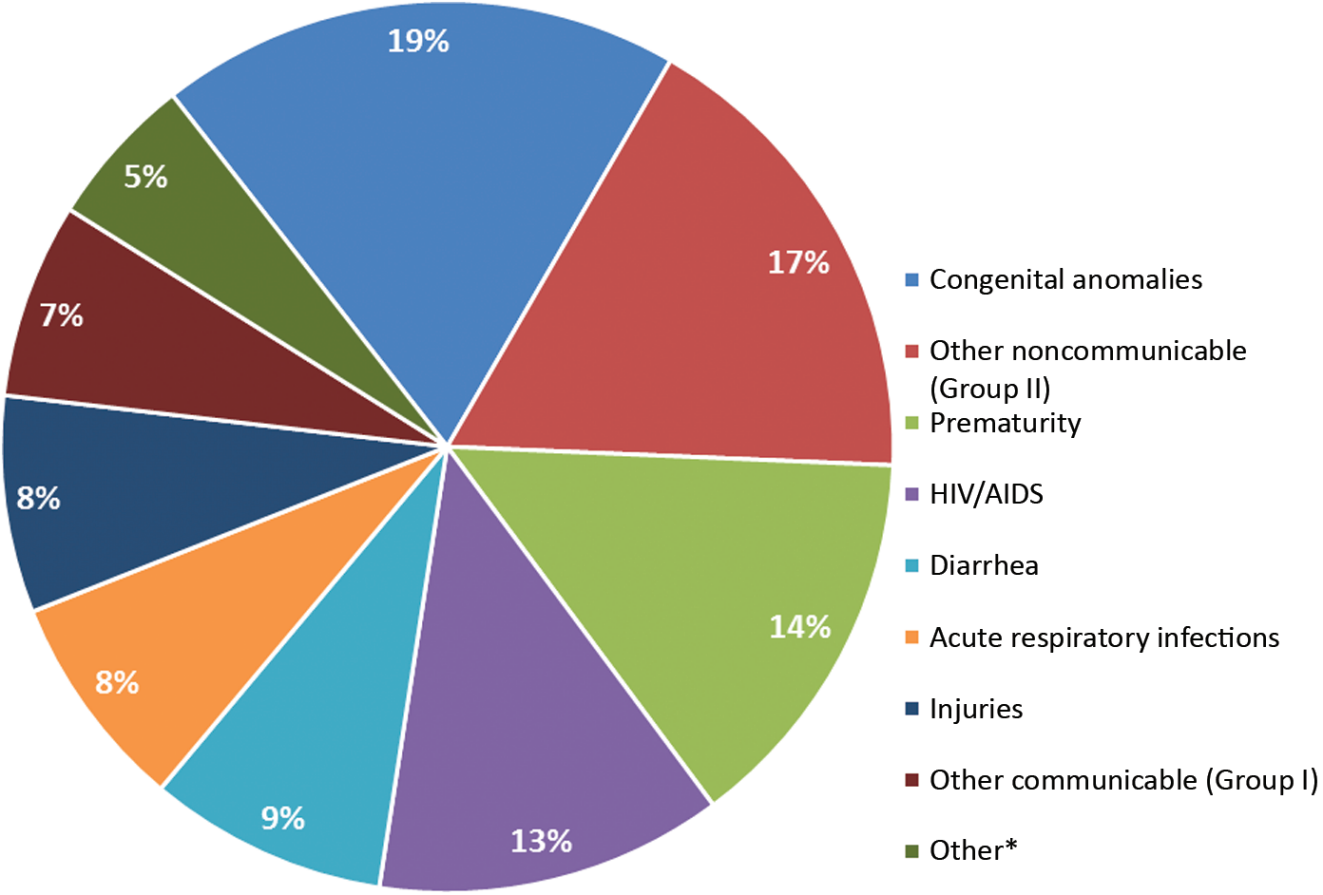
Percentage of deaths based on the United Nations Inter-agency Group for Child Mortality Estimation underlying cause of death categories [12] in children aged 1 month to 14 years in Soweto, South Africa. *Other underlying causes of death include sepsis, birth asphyxia and trauma, ill defined, meningitis/encephalitis. Abbreviation: HIV, human immunodeficiency virus.

Using the findings from the molecular testing, microbiology, and histopathology in addition to the antemortem clinical findings, the DeCoDe panel assigned a CoD in 99% of cases. Details of the certainty of diagnoses for the different underlying CoD and immediate CoD categories are reported in Supplementary Tables 1 and 2. The DeCoDe panel was confident (level 1) for the majority of underlying (81.1%) and immediate CoD (77.2%) diagnoses. Overall, the UN-IGME categories for underlying CoD were 55.1% due to communicable diseases, maternal, perinatal, and nutritional conditions (group 1), 36% due to noncommunicable diseases (group 2), and 8% due to injuries (group 3) (Tables 2 and 3; Figure 2). In group I, these included deaths attributed to underlying HIV/AIDS (22.9%), diarrheal disease (15.7%), acute respiratory infections (14.3%), and complications of prematurity (14.2%).

**Table 2.** Underlying and Immediate Cause of Death Attribution in Infants (1–11 Months of Age), Children (12–59 Months of Age), and Older Children (60 Months–14 Years of Age)

Global Burden of Disease Category^a^	Total (N = 127)	Infants (n = 67)	Children (n = 37)	Older Children (n = 23)
Group I (communicable, maternal, perinatal, and nutritional conditions):	70 (55.1)	43 (64.2)	21 (56.8)	6 (26.1)
Acute respiratory infections	10 (7.9)	4 (6.0)	6 (16.2)	0 (0.0)
Community-acquired pneumonia^b^	8 (6.3)	3 (4.5)	5 (13.5)	0 (0.0)
Myocarditis	1 (0.8)	1 (1.5)	0 (0.0)	0 (0.0)
Rotavirus enteritis^c^	1 (0.8)	0 (0.0)	1 (2.7)	0 (0.0)
Birth asphyxia and trauma	2 (1.6)	1 (1.5)	1 (2.7)	0 (0.0)
Nosocomial pneumonia	1 (0.8)	0 (0.0)	1 (2.7)	0 (0.0)
Hyperosmolality and hypernatremia	1 (0.8)	1 (1.5)	0 (0.0)	0 (0.0)
Diarrhea	11 (8.7)	6 (9.0)	4 (10.8)	1 (4.4)
Gastroenteritis	10 (7.8)	6 (8.8)	4 (10.8)	0 (0.0)
Sepsis	1 (0.8)	0 (0.0)	0 (0.0)	1 (4.4)
HIV/AIDS	16 (12.6)	9 (13.4)	4 (10.4)	3 (13.0)
Community-acquired pneumonia	6 (4.7)	4 (6.0)	1 (2.7)	1 (4.4)
Nosocomial-acquired pneumonia	1 (0.8)	0 (0.0)	1 (2.7)	0 (0.0)
Community-acquired sepsis	2 (1.6)	2 (3.0)	0 (0.0)	0 (0.0)
Nosocomial-acquired sepsis	2 (1.6)	2 (3.0)	0 (0.0)	0 (0.0)
Gastroenteritis	1 (0.8)	1 (1.5)	0 (0.0)	0 (0.0)
Meningitis	1 (0.8)	0 (0.0)	0 (0.0)	1 (4.4)
Encephalitis	1 (0.8)	0 (0.0)	1 (2.7)	0 (0.0)
Heart failure	1 (0.8)	0 (0.0)	0 (0.0)	1 (4.4)
Miliary tuberculosis	1 (0.8)	0 (0.0)	1 (2.7)	0 (0.0)
Meningitis/encephalitis	1 (0.8)	0 (0.0)	1 (2.7)	0 (0.0)
Meningitis	1 (0.8)	0 (0.0)	1 (2.7)	0 (0.0)
Prematurity	18 (14.2)	18 (26.9)	0 (0.0)	0 (0.0)
Community-acquired pneumonia	4 (3.2)	4 (7.5)	0 (0.0)	0 (0.0)
Nosocomial-acquired pneumonia	5 (3.9)	5 (7.5)	0 (0.0)	0 (0.0)
Community-acquired sepsis	1 (0.8)	1 (1.5)	0 (0.0)	0 (0.0)
Nosocomial-acquired sepsis	4 (3.2)	4 (6.0)	0 (0.0)	0 (0.0)
Gastroenteritis	1 (0.8)	1 (1.5)	0 (0.0)	0 (0.0)
Intracranial hemorrhage	1 (0.8)	1 (1.5)	0 (0.0)	0 (0.0)
Necrotizing enterocolitis	1 (0.8)	1 (1.5)	0 (0.0)	0 (0.0)
Acute interstitial pneumonitis (noninfective)	1 (0.8)	1 (1.5)	0 (0.0)	0 (0.0)
Sepsis	3 (2.4)	2 (3.0)	0 (0.0)	1 (4.4)
Sepsis (all)	3 (2.4)	2 (3.0)	0 (0.0)	1 (4.4)
Nosocomial sepsis	1 (0.8)	1 (1.5)	0 (0.0)	0 (0.0)
Other Group I^d^:	9 (7.1)	3 (4.5)	5 (13.5)	1 (4.4)
Community-acquired pneumonia	1 (0.8)	1 (1.5)	0 (0.0)	0 (0.0)
Nosocomial-acquired sepsis	1 (0.8)	0 (0.0)	1 (2.7)	0 (0.0)
Pulmonary tuberculosis	2 (1.6)	1 (1.5)	1 (2.7)	0 (0.0)
Meningitis	1 (0.8)	0 (0.0)	1 (2.7)	0 (0.0)
Encephalitis	1 (0.8)	0 (0.0)	0 (0.0)	1 (4.4)
Viral hepatitis	1 (0.8)	0 (0.0)	1 (2.7)	0 (0.0)
Acute hepatic failure	2 (1.6)	1 (1.5)	1 (2.7)	0 (0.0)
Group II (noncommunicable diseases):	46 (36.2)	22 (32.8)	10 (27.0)	14 (60.9)
Congenital anomalies	24 (18.9)	17 (25.4)	6 (16.2)	1 (4.4)
Community-acquired pneumonia	9 (7.1)	6 (10.0)	3 (8.1)	0 (0.0)
Nosocomial-acquired pneumonia^e^	2 (1.6)	2 (3.0)	0 (0.0)	0 (0.0)
Community-acquired sepsis	2 (1.6)	2 (3.0)	0 (0.0)	0 (0.0)
Nosocomial-acquired sepsis	3 (2.4)	2 (3.0)	1 (2.7)	0 (0.0)
Meningitis (nosocomial)	1 (0.8)	1 (1.5)	0 (0.0)	0 (0.0)
Congenital malformation (tetralogy of Fallot)	1 (0.8)	1 (1.5)	0 (0.0)	0 (0.0)
Gastrointestinal hemorrhage	2 (1.6)	1 (1.5)	1 (2.7)	0 (0.0)
Hemorrhage, lung	1 (0.8)	1 (1.5)	0 (0.0)	0 (0.0)
Congestive heart failure^f^	1 (0.8)	1 (1.5)	0 (0.0)	0 (0.0)
Aspiration pneumonitis	2 (1.6)	0 (0.0)	1 (2.7)	1 (4.4)
Other Group II:	22 (17.3)	5 (7.5)	4 (10.8)	13 (56.5)
Community-acquired pneumonia	4 (3.2)	2 (3.0)	0 (0.0)	2 (8.7)
Nosocomial-acquired pneumonia	1 (0.8)	1 (1.5)	0 (0.0)	0 (0.0)
Nosocomial-acquired sepsis	2 (1.6)	0 (0.0)	1 (2.7)	1 (4.4)
Pulmonary mucormycosis	1 (0.8)	0 (0.0)	0 (0.0)	1 (4.4)
Community-acquired meningitis	1 (0.8)	0 (0.0)	0 (0.0)	1 (4.4)
Nosocomial-acquired meningitis	1 (0.8)	0 (0.0)	0 (0.0)	1 (4.4)
Intracranial abscess	1 (0.8)	0 (0.0)	0 (0.0)	1 (4.4)
Status epilepticus	1 (0.8)	0 (0. 0)	0 (0.0)	1 (4.4)
Pulmonary embolism	1 (0.8)	0 (0.0)	0 (0.0)	1 (4.4)
Heart failure	2 (1.6)	0 (0.0)	2 (5.4)	0 (0.0)
Cerebral infarction	1 (0.8)	0 (0.0)	1 (2.7)	0 (0.0)
Bronchiolitis	1 (0.8)	1 (1.5)	0 (0.0)	0 (0.0)
Acute respiratory distress syndrome	1 (0.8)	0 (0.0)	0 (0.0)	1 (4.4)
Acute hepatic failure	2 (1.6)	1 (1.5)	0 (0.0)	1 (4.4)
Kidney failure	1 (0.8)	0 (0.0)	0 (0.0)	1 (4.4)
Misadventure during surgical/medical care	1 (0.8)	0 (0.0)	0 (0.0)	1 (4.4)
Group III (injuries):	10 (7.9)	1 (1.5)	6 (16.2)	3 (13.0)
Injuries	10 (7.9)	1 (1.5)	6 (16.2)	3 (13.0)
Nosocomial-acquired pneumonia^g^	1 (0.8)	0 (0.0)	1 (2.7)	0 (0.0)
Community-acquired sepsis	1 (0.8)	0 (0.0)	1 (2.7)	1 (4.4)
Nosocomial-acquired sepsis	3 (2.4)	0 (0.0)	2 (5.4)	1 (4.4)
Acute hepatic failure	1 (0.8)	0 (0.0)	1 (2.7)	0 (0.0)
Asphyxiation	1 (0.8)	1 (1.5)	0 (0.0)	0 (0.0)
Poisoning	2 (1.6)	1 (1.5)	0 (0.0)	1 (4.4)
Drowning	1 (0.8)	0 (0.0)	0 (0.0)	1 (4.4)
Ill defined	1 (0.8)	1 (1.5)	0 (0.0)	0 (0.0)

Data are presented as No. (%). Main row headings indicate the underlying cause of death, with immediate causes of death below.

Abbreviation: HIV, human immunodeficiency virus.

^a^Categorized according to the United Nations Inter-agency Group on Child Mortality Estimation classification categories [12].

^b^One pneumonia case had HIV lymphoid interstitial pneumonitis as a coimmediate cause of death.

^c^This case of rotavirus gastroenteritis also had a pneumonia as a coimmediate cause of death.

^d^The underlying conditions in infants included neonatal jaundice from other hepatocellular damage (n = 1), pneumocystosis (n = 1), and tuberculosis (n = 1). The underlying conditions in children included hepatitis A with coma (n = 2), protein energy malnutrition, unspecified (n = 1), and tuberculosis (n = 2). The underlying condition in older children included chickenpox (varicella) (n = 1).

^e^One case of nosocomial *Pseudomonas* pneumonia also had nosocomial *Escherichia coli* meningitis as a coimmediate cause of death; the immediate cause of death in this case is recorded as meningitis.

^f^This case of congestive heart failure also had congenital hypoplasia and dysplasia of the lung as a coimmediate cause of death.

^g^This case of nosocomial *Staphylococcus aureus* pneumonia also had nosocomial disseminated herpes simplex virus infection as a coimmediate cause of death.

**Table 3.** Underlying Cause of Death Categories and Specific Underlying Cause of Death Attribution in Infants (0–11 Months of Age), Children (12–59 Months of Age), and Older Children (60 Months–14 Years of Age)

Global Burden of Disease Category	Total	Infants	Children	Older Children
	(N = 127)	(n = 67)	(n = 37)	(n = 23)
Group I (communicable, maternal, perinatal, and nutritional conditions)^a^:	70 (55.1)	43 (64.2)	21 (56.8)	6 (26.1)
Acute respiratory infections	10 (7.9)	4 (6.0)	6 (16.2)	0 (0.0)
Pneumonia	9 (7.1)	3 (4.5)	6 (16.2)	0 (0.0)
Acute bronchiolitis	1 (0.8)	1 (1.5)	0 (0.0)	0 (0.0)
Birth asphyxia and trauma	2 (1.6)	1 (1.5)	1 (2.7)	0 (0.0)
Birth asphyxia, unspecified	2 (1.6)	1 (1.5)	1 (2.7)	0 (0.0)
Diarrhea	11 (8.7)	6 (9.0)	4 (10.8)	1 (4.4)
HIV/AIDS	16 (12.6)	9 (13.4)	4 (10.8)	3 (13.0)
HIV disease	15 (11.8)	9 (13.4)	3 (8.1)	3 (13.0)
HIV disease resulting in wasting syndrome	1 (0.8)	0 (0.0)	1 (2.7)	0 (0.0)
Meningitis/encephalitis	1 (0.8)	0 (0.0)	1 (2.7)	0 (0.0)
Sepsis	3 (2.4)	2 (3.0)	0 (0.0)	1 (4.4)
Prematurity complications	18 (14.2)	18 (26.9)	0 (0.0)	0 (0.0)
Other Group I:	9 (7.1)	3 (4.5)	5 (13.5)	1 (4.4)
Congenital rubella syndrome	1 (0.8)	1 (1.5)	0 (0.0)	0 (0.0)
Chickenpox (varicella)	1 (0.8)	0 (0.0)	0 (0.0)	1 (4.4)
Hepatitis A with coma	2 (1.6)	0 (0.0)	2 (5.4)	0 (0.0)
Neonatal jaundice from other hepatocellular damage	1 (0.8)	1 (1.5)	0 (0.0)	0 (0.0)
Pneumocystosis	1 (0.8)	1 (1.5)	0 (0.0)	0 (0.0)
Protein energy malnutrition, unspecified	1 (0.8)	0 (0.0)	1 (2.7)	0 (0.0)
Tuberculosis	3 (2.4)	1 (1.5)	2 (5.4)	0 (0.0)
Group II (noncommunicable diseases)^b^:	46 (36.2)	22 (32.8)	10 (27.0)	14 (60.9)
Congenital anomalies	24 (18.9)	17 (25.4)	6 (16.2)	1 (4.4)
Cardiovascular system abnormalities^c^	4 (3.1)	2 (3.0)	2 (5.4)	0 (0.0)
Charge syndrome	1 (0.8)	1 (1.5)	0 (0.0)	0 (0.0)
Chromosomal abnormality, unspecified	1 (0.8)	1 (1.5)	0 (0.0)	0 (0.0)
Congenital hydrocephalus	1 (0.8)	0 (0.0)	0 (0.0)	1 (4.4)
Congenital malformation syndromes predominantly affecting facial appearance	1 (0.8)	1 (1.5)	0 (0.0)	0 (0.0)
Down syndrome, unspecified	6 (4.7)	5 (7.5)	1 (2.7)	0 (0.0)
GI tract abnormalities^d^	9 (7.1)	7 (10.4)	2 (5.4)	0 (0.0)
Spina bifida with hydrocephalus, unspecified	1 (0.8)	0 (0.0)	1 (2.7)	0 (0.0)
Other Group II:	22 (17.3)	5 (7.5)	4 (10.8)	13 (56.5)
Cerebral infarction	1 (0.8)	0 (0.0)	1 (2.7)	0 (0.0)
Cerebral palsy	2 (1.6)	2 (3.0)	0 (0.0)	0 (0.0)
Chronic kidney disease	1 (0.8)	0 (0.0)	0 (0.0)	1 (4.4)
Coagulation defect (other)	1 (0.8)	0 (0.0)	0 (0.0)	1 (4.4)
Fanconi anemia	1 (0.8)	0 (0.0)	0 (0.0)	1 (4.4)
Epilepsy	1 (0.8)	0 (0.0)	0 (0.0)	1 (4.4)
Galactosemia	1 (0.8)	1 (1.5)	0 (0.0)	0 (0.0)
Inflammatory liver disease, unspecified	1 (0.8)	0 (0.0)	0 (0.0)	1 (4.4)
Kidney failure, unspecified	1 (0.8)	0 (0.0)	0 (0.0)	1 (4.4)
Malignancies^e^	9 (7.1)	2 (3.0)	2 (5.4)	5 (21.7)
Mastoiditis, unspecified	1 (0.8)	0 (0.0)	0 (0.0)	1 (4.4)
Metabolic disorder, unspecified	1 (0.8)	1 (1.5)	0 (0.0)	0 (0.0)
Parvovirus cardiomyopathy	1 (0.8)	0 (0.0)	1 (2.7)	0 (0.0)
Group III (injuries)^f^:	10 (7.9)	1 (1.5)	6 (16.2)	3 (13.0)
Injuries	10 (7.9)	1 (1.5)	6 (16.2)	3 (13.0)
Asphyxiation	1 (0.8)	1 (1.5)	0 (0.0)	0 (0.0)
Burns	4 (3.1)	0 (0.0)	4 (10.8)	0 (0.0)
Drowning	1 (0.8)	0 (0.0)	0 (0.0)	1 (4.4)
Motor vehicle accident (pedestrian)	1 (0.8)	0 (0.0)	0 (0.0)	1 (4.4)
Organophosphate poisoning	2 (1.6)	0 (0.0)	1 (2.7)	1 (4.4)
Poisoning, drugs	1 (0.8)	0 (0.0)	1 (2.7)	0 (0.0)
Ill defined^g^	1 (0.8)	1 (1.5)	0 (0.0)	0 (0.0)

Data are presented as No. (%). Main row headings indicate the underlying cause of death, with immediate causes of death below.

Abbreviations: GI, gastrointestinal; HIV, human immunodeficiency virus.

^a^Communicable, maternal, perinatal, and nutritional conditions includes those conditions whose *International Classification of Diseases, Tenth Revision* (*ICD-10*) code is among the following: A00–B99, D50–D53, D64.9, E00–E02, E40–E64, G00–G09, H65–H66, J00–J22, J85, N30, N34, N390, N70–N73, O00–P96, U04 [12].

^b^Noncommunicable conditions are those conditions whose *ICD-10* code falls into the any of the following codes: C00–C97, D00–D48, D55–D64 (exclude D64.9), D65–D89, E03–E34, E65–E88, F01–F99, G10–G98, H00–H61, H68–H93, I00–I99, J30–J84, J86–J98, K00–K92, L00–L98, M00–M99, N00–N28, N31–N32, N35–N64 (exclude N39.0), N75–N98, Q00–Q99 [12].

^c^Cardiovascular system abnormalities included 1 case each of tetralogy of Fallot (infant), discordant ventriculoarterial connection (child), hypoplasia of aorta (child) and congenital malformation of the heart, unspecified (infant).

^d^The GI tract abnormalities included 3 cases of atresia of bile ducts; 2 cases of congenital absence, atresia and stenosis of jejunum; and 1 case each of gastroschisis, Hirschsprung disease, congenital absence, atresia and stenosis of anus without fistula, and congenital absence, atresia and stenosis of duodenum. All these abnormalities were in the infant group except 2 cases of atresia of bile ducts (child).

^e^The malignancies identified included 2 cases each of acute lymphoblastic leukemia (1 child and 1 older child) and acute myeloblastic leukemia (1 child and 1 older child); and 1 case each of benign neoplasm of unspecified adrenal gland (older child); Hodgkin lymphoma, unspecified (older child); non-Hodgkin lymphoma, unspecified (older child); Burkitt lymphoma (older child) and malignant neoplasm of brain, unspecified (infant).

^f^Group III category includes all conditions whose *ICD-10* code is included in V01–Y89 [12].

^g^Ill-defined refers to a cause of death which could not be determined using the available evidence.

**Figure 3.**
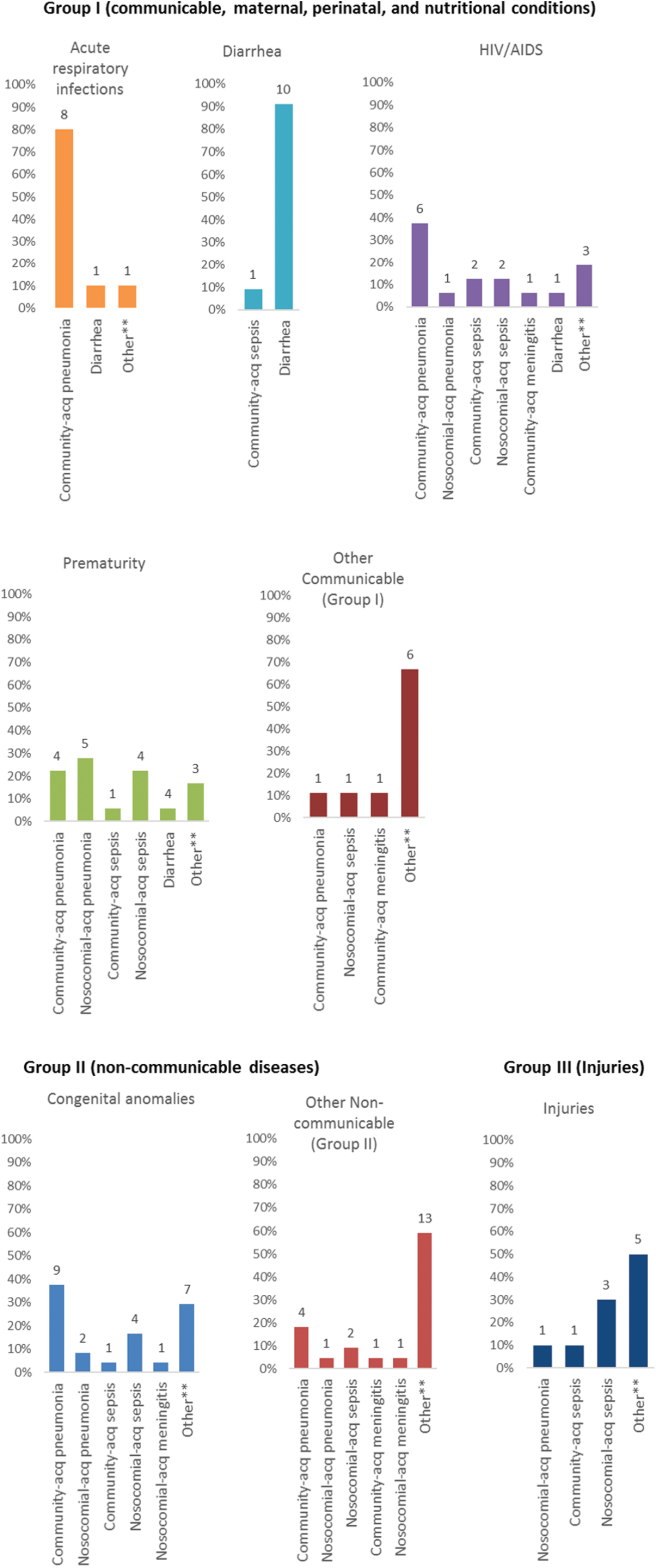
Underlying causes of death according to the United Nations Inter-agency Group for Child Mortality Estimation global burden of disease categories [12] and the proportion of the infections as the immediate cause of death (CoD) for the given underlying CoD categories. **Other immediate CoD (excluding pneumonia, sepsis, meningitis, and diarrhea) includes other circulatory, hepatic failure, pulmonary tuberculosis, encephalitis, aspiration pneumonia, gastrointestinal hemorrhage, poisoning, hepatitis, pulmonary mucormycosis, hyperosmolality, intracranial abscess, status epilepticus, pulmonary embolism, acute respiratory distress syndrome, acute interstitial pneumonitis, kidney failure, necrotizing enterocolitis, tetralogy of Fallot, hemorrhage from respiratory passage, sudden infant death syndrome, asphyxiation, drowning, and complications during surgery. Abbreviations: acq, acquired; HIV, human immunodeficiency virus.

Among cases with HIV as the underlying CoD (n = 16), infection was the immediate CoD in 15 (93.8%) cases, mainly from pneumonia and sepsis (n = 7 [43.8%] and n = 4 [25.0%], respectively; Figure 3; Table 2). Similarly, infections were also the immediate CoD in 83.3% of 18 deaths attributed to complications of prematurity as the underlying cause, also mainly due to pneumonia (n = 9 [50.0%]) or sepsis (n = 5 [27.8%]) (Figure 3; Table 2).

Among the 10 cases with acute respiratory infections as the underlying CoD, 8 deaths were due to the pneumonia caused by RSV (n = 2), *Streptococcus pneumoniae* (n = 2), *Haemophilus influenzae* (n = 1), *Staphylococcus aureus* (n = 1), *P. jirovecii* (n = 1), and influenza A virus (n = 1). Similarly, 10 of 11 (90.9%) deaths attributed to diarrhea as the underlying cause died as a consequence thereof, whereas 1 child died of community-acquired *Escherichia coli* sepsis. The diarrhea deaths were due to enteroinvasive *E. coli/Shigella* (n = 4) and norovirus (n = 1), while in 6 cases no pathogen was identified. Details of other group 1 underlying CoD are outlined in Table 3 and included 3 (2.4%) deaths due to tuberculosis, 2 (1.6%) due to hepatitis A, and 1 each from varicella and congenital rubella syndrome.

The majority of UN-IGME group II (n = 46) category deaths were attributed to congenital malformations (n = 24 [18.9% of overall deaths]) of which 70.8% were in infants and 25.0% in children (Table 3). The most frequent congenital abnormalities were gastrointestinal malformations (37.5%), Trisomy-21 (25%), and cardiovascular system malformations (25%) (Table 3). Infections were also the dominant immediate CoD among cases with underlying congenital abnormalities, including 45.8% (n = 11) pneumonia, 20.8% (n = 5) sepsis, and 4.2% (n = 1) meningitis (Figure 3; Table 2). Among these infection-related deaths (n = 17), 58.8% (n = 10) were community-associated, and 41.2% (n = 7) were hospital-acquired. Malignancies were diagnosed as an underlying CoD in 7.1% of case overall, and contributed to 40.9% (19/43) of UN-IGME group II deaths (Table 2).

The majority of deaths classified as UN-IGME group III (n = 10) occurred in children (60%) and older children (30%). These included deaths from burns (n = 4 [40.0%]), poisoning (n = 3 [30.0%]), foreign body asphyxiation (n = 1 [10.0%]), drowning (n = 1 [10.0%]), and motor vehicle accident (n = 1 [10.0%]) (Table 3). Among the group III deaths, hospital-acquired infection was the immediate CoD in 40.0% of cases, and community-acquired infection in 10.0% (Figure 3; Table 2).

### Overall Contribution of Infections Either as an Immediate or Underlying Cause of Death

Overall, infections were attributed as an underlying or immediate cause in 78% (n = 99) of deaths. This included 48 of 127 (37.8%) cases where infection was an underlying CoD, and 51 cases where the underlying CoD was a noninfectious medical condition (64.6% of 79).

Pneumonia was the commonest infection-related CoD (44% [n = 44/99]), 73% (n = 32/44) of which were community-associated and 27% (n = 12/44) hospital-acquired (Table 4). Among community-associated pneumonia cases (n = 32), RSV was the most commonly implicated pathogen (21.9% [n = 7]) and occurred exclusively among infants, including 4 cases in children with a noninfectious underlying CoD. Other pathogens causing community-acquired pneumonia deaths were *P. jirovecii* (18.8% [n = 6]), including 3 in HIV-infected children and 3 in infants without HIV infection, 1 of whom had underlying cerebral palsy, another was HIV exposed; the third was HIV unexposed and nutritional status was unknown. Other organisms implicated in community-acquired pneumonia deaths included *K. pneumoniae* (15.6% [n = 5]), cytomegalovirus (CMV; 15.6% [n = 5]), influenza virus (12.5% [n = 4]), and *S. pneumoniae* (12.5% [n = 4]). Forty percent (n = 2/5) of CMV pneumonitis– and 25% (n = 1/4) of pneumococcal pneumonia–attributed deaths were in HIV-infected children.

**Table 4.** Pathogens Identified in Children in Whom the Immediate or Underlying Cause of Death Was Infection Related, Stratified by Disease Syndrome

Diagnosis and Pathogen	Overall (N = 127)	<12 mo (n = 67)	12–59 mo (n = 37)	≥60 mo (n = 23)
Community-associated pneumonia^a^	32 (25.2)	20 (29.9)	10 (27.0)	2 (8.7)
RSV	7 (5.5)	7 (10.5)	0 (0.0)	0 (0.0)
*Pneumocystis jirovecii*	6 (4.7)	6 (9.0)	0 (0.0)	0 (0.0)
CMV	5 (3.9)	5 (7.5)	0 (0.0)	0 (0.0)
*Klebsiella pneumoniae*	5 (3.9)	3 (4.5)	2 (5.4)	0 (0.0)
Influenza virus	4 (3.1)	1 (1.5)	2 (5.4)	1 (4.4)
*Streptococcus pneumoniae*	4 (3.1)	0 (0.0)	4 (10.8)	0 (0.0)
*Moraxella catarrhalis*	3 (2.4)	1 (1.5)	2 (5.4)	0 (0.0)
*Haemophilus influenzae*	3 (2.4)	0 (0.0)	3 (8.1)	0 (0.0)
*Bordetella pertussis*	2 (1.6)	2 (3.0)	0 (0.0)	0 (0.0)
*Pseudomonas aeruginosa*	2 (1.6)	1 (1.5)	1 (2.7)	0 (0.0)
*Staphylococcus aureus*	2 (1.6)	1 (1.5)	1 (2.7)	0 (0.0)
HMPV	1 (0.8)	1 (1.5)	0 (0.0)	0 (0.0)
*Streptococcus* spp (other)	1 (0.8)	0 (0.0)	1 (2.7)	0 (0.0)
Unspecified	1 (0.8)	0 (0.0)	0 (0.0)	1 (4.4)
Nosocomial pneumonia^b^	12 (9.4)	9 (13.4)	3 (8.1)	0 (0.0)
*K. pneumoniae*	8 (6.3)	6 (9.0)	2 (5.4)	0 (0.0)
*S. aureus*	2 (1.6)	1 (1.5)	1 (2.7)	0 (0.00)
*Acinetobacter baumannii*	1 (0.8)	1 (1.5)	0 (0.0)	0 (0.0)
HMPV	1 (0.8)	0 (0.0)	1 (2.7)	0 (0.0)
*P. aeruginosa*	1 (0.8)	1 (1.5)	0 (0.0)	0 (0.0)
RSV	1 (0.8)	1 (1.5)	0 (0.0)	0 (0.0)
Community-associated sepsis	7 (5.5)	4 (6.0)	1 (2.7)	2 (8.7)
*Escherichia coli*	4 (3.1)	2 (3.0)	1 (2.7)	1 (4.4)
*S. aureus*	1 (0.8)	0 (0.0)	0 (0.0)	1 (4.4)
Unspecified	2 (1.6)	2 (3.0)	0 (0.0)	0 (0.0)
Nosocomial sepsis^c^	17 (13.4)	9 (13.4)	5 (13.5)	3 (13.1)
*A. baumannii*	8 (6.3)	5 (7.5)	2 (5.4)	1 (4.4)
*K. pneumoniae*	6 (4.7)	3 (4.5)	2 (5.4)	1 (4.4)
*S. aureus*	3 (2.4)	1 (1.5)	2 (5.4)	0 (0.0)
*E. coli*	2 (1.6)	2 (3.0)	0 (0.00)	0 (0.0)
*Candida parapsilosis*	1 (0.8)	1 (1.5)	0 (0.0)	0 (0.0)
*Clostridium* spp	1 (0.8)	0 (0.0)	0 (0.0)	1 (4.4)
*Enterococcus faecalis*	1 (0.8)	0 (0.0)	1 (2.7)	0 (0.0)
*P. aeruginosa*	1 (0.8)	0 (0.0)	1 (2.7)	0 (0.0)
Gastroenteritis	14 (11.0)	8 (11.9)	5 (13.5)	1 (4.4)
*Salmonella*	1 (0.8)	1 (1.5)	0 (0.0)	0 (0.0)
Enteroinvasive *E. coli/Shigella*^d^	4 (3.1)	1 (1.5)	2 (5.4)	1 (4.4)
Rotavirus enteritis^e^	1 (0.8)	0 (0.0)	1 (2.7)	0 (0.0)
Norovirus	2 (1.6)	2 (3.0)	0 (0.00)	0 (0.00)
No pathogen attributed	6 (4.7)	4 (6.0)	2 (5.4)	0 (0.0)
Community-associated meningitis^f^	4 (3.1)	0 (0.0)	2 (5.4)	2 (8.7)
*S. pneumoniae*	2 (1.6)	0 (0.0)	1 (2.7)	1 (4.4)
*E. coli*	1 (0.8)	0 (0.0)	0 (0.0)	1 (4.4)
*Neisseria meningitidis*	1 (0.8)	0 (0.0)	0 (0.0)	1 (4.4)
*Mycobacterium tuberculosis*	1 (0.8)	0 (0.0)	1 (2.7)	0 (0.0)
Nosocomial meningitis^g^	2 (1.6)	1 (1.5)	0 (0.0)	1 (4.4)
*A. baumannii*	1 (0.8)	0 (0.0)	0 (0.0)	1 (4.4)
*Candida albicans*	1 (0.8)	0 (0.0)	0 (0.0)	1 (4.4)
*E. coli*	1 (0.8)	1 (1.5)	0 (0.0)	0 (0.0)
Other infection	4 (3.1)	3 (34.5)	1 (2.7)	0 (0.0)
CMV^h^	2 (1.6)	2 (3.0)	0 (0.0)	0 (0.0)
HSV (nosocomial)	1 (0.8)	0 (0.0)	1 (2.7)	0 (0.0)
Rubella (congenital)	1 (0.8)	1 (1.5)	0 (0.00)	0 (0.00)

Data are presented as No. (%).

Abbreviations: CMV, cytomegalovirus; HMPV, human metapneumovirus; HSV, herpes simplex virus; RSV, respiratory syncytial virus.

^a^The total is less than the column sum because of the following coinfections: *H. influenzae*, *S. pneumoniae*, and influenza A virus (underlying); *K. pneumoniae* and *M. catarrhalis*; *P. jirovecii* and *M. catarrhalis*; *P. jiroveci* and CMV; *S. aureus* (methicillin resistant) and other *Streptococcus* spp; *P. jiroveci* and RSV; *H. influenzae* and *S. pneumoniae*; *K. pneumoniae* and influenza C virus; *P. aeruginosa* and *S. pneumoniae*; *K. pneumoniae* and RSV; *H. influenzae* and *M. catarrhalis*.

^b^The total is less than the column sum because of the following coinfections attributed to pneumonia cases: *K. pneumoniae* and HMPV; *K. pneumoniae* and RSV.

^c^The total is less than the column sum because of the following coinfections among sepsis cases: *S. aureus* and *P. aeruginosa*; *A. baumannii* sepsis and *S. aureus* (methicillin resistant); *K. pneumoniae* and *A. baumannii*; *A. baumannii* and *E. coli*; *K. pneumoniae* and *E. faecalis*; *A. baumannii* and *S. aureus*; *A. baumannii* and *C. parapsilosis*.

^d^One case of enteroinvasive *E. coli/Shigella* gastroenteritis as underlying cause of death (CoD) had *E. coli* sepsis as the immediate CoD.

^e^This case had of rotavirus gastroenteritis also had influenza virus pneumonia as a coimmediate CoD.

^f^The total is less than the column sum because of the following coinfections: *S. pneumoniae* meningitis and *N. meningitidis* meningitis.

^g^The total is less than the column sum because of 1 case with *A. baumannii* and *C. albicans* meningitis.

^h^Although the 2 cases of disseminated CMV were community-acquired, they had coinfections with nosocomial *A. baumannii* sepsis and nosocomial *K. pneumoniae* sepsis.

*Klebsiella pneumoniae* was the commonest (66.7% [n = 8/12]) pathogen causing hospital-acquired pneumonia deaths, and all cases had a noninfectious underlying CoD. Seventy-one percent (n = 17/24) of the sepsis-related deaths were hospital-acquired, most commonly due to *Acinetobacter baumannii* (47.1% [n = 8/17]), *K. pneumoniae* (35.3% [n = 6/17]), and *S. aureus* (17.6% [n = 3/17]). Among the 7 community-acquired sepsis deaths, *E. coli* accounted for 57.1% (n = 4/7) of cases and *S. aureus* for 14.3% (n = 1/7); no pathogen was identified in 2 cases (Table 4).

Of the 6 deaths due to meningitis as the immediate cause, 4 were community-acquired (*S. pneumoniae*; *Neisseria meningitidis* and *S. pneumoniae* coinfection; *E. coli*; and *Mycobacterium tuberculosis*); and 2 hospital-acquired cases were due to *A. baumannii* and *Candida albicans* coinfection (n = 1) and *E. coli* (n = 1).

## DISCUSSION

This pilot study demonstrates the value of MITS, interpreted with antemortem clinical data, in attributing highly specific causes of death in an LMIC setting where under-5 mortality rate (per 1000 live births) was estimated to be 53 in 2013 (unpublished data); compared to the national estimate of 42 per 1000 in 2015 [2]. The DeCoDe panel, with generally high level of confidence, attributed an underlying and immediate CoD in nearly all (99%) cases. Furthermore, for deaths associated with infection, a specific pathogen was identified in 96% of cases. Elucidating such granular information on the causes of death could be extremely useful for planning and prioritizing future interventions aimed to reduce childhood mortality, even when the underlying cause itself might not be preventable (eg, premature birth).

Notably, this pilot, proof-of-concept study was undertaken as a prelude to the multicountry Child Health and Mortality Prevention Surveillance (CHAMPS) program to establish the acceptability and added value of MITS in providing granular detail on the causes of childhood death [13]. The leading underlying CoD among our study population were classified under UN-IGME group I, the most common of which were prematurity (28%), HIV/AIDS (20%), diarrheal disease (16%), and acute respiratory infections (16%). The respective national estimates for these conditions as underlying cause of death among South African children aged 1–59 months in 2015 were 0.7%, 1.7%, 14.5%, and 12.6%, respectively [14, 15]. Caveats of comparing our study results to the national estimates on CoD include that deaths investigated in our study excluded deaths not presenting or occurring at the facility, as well as biases introduced from the phasing-in of enrollment in the different nonmedical wards at the hospital during the course of the study.

Consequently, differences in the percentage of deaths attributed to prematurity and HIV/AIDS as the underlying CoD in our study, compared with the national modeled estimates, could be due to biased case enrollment in our study. Alternately, it could be reflective of inaccuracies in the current modeling approaches of CoD attribution. Interestingly, however, the proportion of deaths attributed to diarrhea and acute respiratory infections as an underlying cause in our study mirrored those of the national estimates [1, 15]. Also, the percentage of deaths attributed to meningitis (1.6%) as an underlying cause in our study was similar to national estimates (1.5%), whereas the respective figure for sepsis (2.4% in our study) as an underlying cause was higher than the national estimate (0.2%) [15].

Despite the above study limitation, our findings provide detailed insight into the specific causes of death, which would otherwise mainly have been analyzed at the syndromic level. This included the dominant role of infections as either the immediate or underlying CoD in children (78%), with specifically identified preventable or treatable organisms contributing to 94% of HIV-related deaths and 90% of pneumonia deaths. The role of infections reflected here excludes those contributing to death as antecedent CoD, and their overall contribution is likely to be even greater when antecedent morbid conditions are considered. Also, notably, only a single death was attributed to severe malnutrition as the underlying cause, although 62% of the children who died were categorized as malnourished.

In our setting where PCV was introduced into the public immunization program in 2009, RSV was the commonest pathogen implicated in respiratory deaths, albeit a limited number of pneumonia cases. This included deaths occurring in prematurely born infants, and children with underlying neoplasms and congenital malformations. Without postmortem investigation of these cases, the role of RSV and other pathogens implicated in pneumonia-associated childhood deaths in our study would be underappreciated.

Significant progress has occurred in reducing mother-to-child HIV transmission in South Africa, as well as providing antiretroviral treatment to all HIV-infected children upon their diagnosis [16, 17]. Nonetheless, we observed pneumonia from *P. jirovecii* (n = 6) and CMV (n = 5) as important causes of death in these children, similar to observations before antiretroviral treatment was standard of care in settings such as ours [18]. This observation suggests deficiencies in the current HIV treatment program in South Africa, where death from preventable causes such as *P. jirovecii* pneumonia is still prevalent in HIV-infected children. The findings indicate the utility of MITS in prompting a review of recommendations and practices to avoid these preventable deaths.

Analysis of the immediate CoD in our study also highlights the contribution of sepsis, which would largely be missed if focusing only on the underlying CoD [19]. Considering that death from sepsis is treatable, knowledge of the commonly implicated pathogens as revealed by MITS could inform empiric antibiotic therapy and sensitize physicians to their important role in childhood death, including the role of hospital-acquired infections. In developing countries, *Klebsiella* species and *S. aureus* have been attributed as important pathogens of hospital-acquired sepsis in infants [20–22]. Our study identified multidrug-resistant (data not shown) *A. baumannii* as the dominant (47.1%) pathogen causing hospital-acquired sepsis. The emerging dominance of multidrug-resistant *Klebsiella* species and *A. baumannii* infections necessitates a review of empiric treatment of hospital-acquired infections in settings such as ours [23–27].

Congenital malformations (22.1%) were the leading underlying CoD among children 1–59 months of age (group II); the proportion is 11-fold higher than national estimates (2%) [15]. The difference might reflect biases in enrollment in our study or, conversely, underascertainment of underlying congenital abnormalities in current CoD modeling exercises. Nevertheless, MITS provided further insight that the majority of deaths in these children were also from treatable or preventable infections (73.9%), with only 21.7% of the deaths in this group attributed directly to the underlying malformation.

Although MITS provided insight into the causal pathway of death in most cases, it might be of limited utility in attributing the underlying CoD in noncommunicable disease such as occult congenital malformations, which we mainly diagnosed based on antemortem clinical information. This highlights the need for a holistic approach in using all available information for fully characterizing the CoD in children. Another limitation of MITS, which systematically samples predefined anatomical regions, is that focal abnormalities could be missed. Also, although we used molecular assays for identifying some organisms, the interpretation thereof could be controversial in the absence of validating the significance of their presence. This was partly addressed in our study by interpreting the finding from the molecular and microbial culture tests, together with the clinical and histopathological results.

In conclusion, our pilot demonstrated that almost two-thirds of screened parents approached for performing MITS on their deceased children consented to study participation. Furthermore, despite the study limitations, including the generalizability of the study findings, we demonstrate the utility of MITS in contributing to a granular understanding of the causal pathway of death in children. Future studies, such as CHAMPS [13], which are designed to be more generalizable to the studied populations, would help inform the prioritization of interventions and research that is required toward achieving the UN Sustainable Development Goal 3.2 target of reducing under-5 childhood death rates (per 1000 live births) from 39.1 in 2017 to 25 by 2030.

## Supplementary Data

Supplementary materials are available at *Clinical Infectious Diseases* online. Consisting of data provided by the authors to benefit the reader, the posted materials are not copyedited and are the sole responsibility of the authors, so questions or comments should be addressed to the corresponding author.

ciz550_suppl_Supplementary_Tables

## Notes

***Acknowledgments.*** The authors acknowledge the following pathologists for their contribution to undertaking diagnostic histopathology investigations: Dr S. Pather, Dr R. Mohanlal, Dr E. van den Berg, Dr R. Wadee, and Ms M. Suleman, all from the National Health Laboratory Service Department of Anatomical Pathology, Faculty of Health Sciences, University of the Witwatersrand. The authors acknowledge the important contribution of the Determining Cause of Death (DeCoDe) panel (speciality in parenthesis) that was chaired by Scott Dowell (epidemiologist, paediatrician) and Christopher Wilson (infectious diseases, pediatrician). Other DeCoDe panel members were Quique Bassat (infectious diseases, pediatrician), Dianna Blau (microbiology), Robert Breiman (epidemiology, infectious diseases, physician), Daniel Chandramohan (epidemiology, World Health Organization *International Classification of Diseases* coding specialist), Richard Chawana (physician), Kevin Clarke (microbiology), Martin Hale (pathology), Penny Heaton (physician), Eric Houpt (microbiology, infectious disease, physician), Aggrey Igunza (study clinician), Grace Irimu (pediatrician), Reinhard Kaiser (epidemiology, physician), Nicholas Kassebaum (microbiology), Keith Klugman (microbiology, infectious diseases, physician), Karen Kotloff (infectious diseases, pediatrician), Shabir Madhi (infectious diseases, pediatrician), Kim Mulholland (infectious diseases, epidemiology, pediatrician), Jaume Ordi (pathology), Robert Pattinson (obstetrician), Rebecca Philipsborn (pediatrician), Pratima Raghunathan (epidemiology), Milagritos Tapia (infectious diseases, pediatrician). Allan Taylor (epidemiology, pediatrician), Sherif Zaki (pathology).

***Disclaimer.*** The findings and conclusions in this report are those of the author(s) and do not necessarily represent the official position of the Centers for Disease Control and Prevention. The funder played no role in the study design, data collection and analyses, preparation, or approval of this manuscript. The Bill & Melinda Gates Foundation staff participated in the DeCoDe panel and the program officer (Scott Dowell) was provided the opportunity to review a preliminary version of this manuscript for factual accuracy, but the authors are solely responsible for final content and interpretation.

***Financial support.*** This work was supported by the Bill & Melinda Gates Foundation (award number OPP1101764).

***Supplement sponsorship.*** This supplement is sponsored by the Emory Global Health Institute and the Bill & Melinda Gates Foundation.

***Potential conflicts of interest.*** S. A. M. has received grants from the Bill & Melinda Gates Foundation, Pfizer, Glaxo Smith Kline, Biovac, Novavax, and Sanofi Pasteur; and honoraria from The Bill & Melinda Gates Foundation. All other authors report no potential conflicts of interest. All authors have submitted the ICMJE Form for Disclosure of Potential Conflicts of Interest. Conflicts that the editors consider relevant to the content of the manuscript have been disclosed.
