# Supplementary material for: Potential of Minimally Invasive Tissue Sampling for Attributing Specific Causes of Childhood Deaths in South Africa: A Pilot, Epidemiological Study
Source: Clin Infect Dis. 2019 Oct 9;69(Suppl 4):S361–73. doi: 10.1093/cid/ciz550 (PMC6785686; doi:10.1093/cid/ciz550)
Supplement: ciz550_suppl_Supplementary_Tables [file ciz550_suppl_supplementary_tables.docx]

**SUPPLEMENTARY TABLES**

**Supplementary Table 1: Level of certainty for underlying cause of death attribution by the panel.**

| **Global Burden of Disease category** | **Total** | **Level of Certainty** | | |
| --- | --- | --- | --- | --- |
|  | **N** | **Level 1^a^**  **(Confident)** | **Level 2^b^**  **(Probable)** | **Level 3^c^**  **(Uncertain)** |
| **Group I (Communicable, maternal, perinatal and nutritional conditions)** | **70 (55.1%)** | **59 (84.3%)** | **9 (12.9%)** | **2 (2.9%)** |
| Acute respiratory infections | 10 (7.9%) | 8 (80.0%) | 2 (20.0%) | 0 (0.0%) |
| Birth asphyxia and trauma | 2 (1.6%) | 0 (0.0%) | 1 (50.0%) | 1 (50.0%) |
| Diarrhoea | 11 (8.7%) | 9 (81.8%) | 2 (18.2%) | 0 (0.0%) |
| HIV/AIDS | 16 (12.6%) | 15 (93.8%) | 1 (6.2%) | 0 (0.0%) |
| Meningitis/encephalitis | 1 (0.8%) | 1 (100.0%) | 0 (0.0%) | 0 (0.0%) |
| Prematurity | 18 (14.2%) | 18 (100.0%) | 0 (0.0%) | 0 (0.0%) |
| Sepsis | 3 (3.4%) | 2 (66.7%) | 0 (0.0%) | 1 (33.3%) |
| Other Group I | 9 (7.1%) | 6 (66.7%) | 3 (33.3%) | 0 (0.0%) |
| **Group II (Noncommunicable diseases)** | **46 (36.2%)** | **39 (84.8%)** | **5 (10.9%)** | **2 (4.4%)** |
| Congenital anomalies | 24 (18.9%) | 20 (83.3%) | 2 (8.3%) | 2 (8.3%) |
| Other Group II | 22 (17.3%) | 19 (86.4%) | 3 (13.6%) | 0 (0.0%) |
| **Group III (Injuries)** | **10 (7.9%)** | **5 (50.0%)** | **3 (30.0%)** | **2 (20.0%)** |
| Injuries | 10 (7.9%) | 5 (50.0%) | 3 (30.0%) | 2 (20.0%) |
| **Ill defined** | **1 (0.8%)** | **0 (0.0%)** | **1 (100.0%)** | **0 (0.0%)** |
| **Total** | **127 (100%)** | **103 (81.1%)** | **18 (14.2%)** | **6 (4.7%)** |

^a^Level 1 certainty was considered where there were severe pathological findings likely to be the cause of death and the presence of microorganisms that represent true pathogens and/or consistent detection of microorganisms in multiple samples.

^b^Level 2 certainty was considered where there were pathological findings of moderate intensity, probably causing death and the presence of microorganisms that can either represent true pathogens or colonizing/contaminants detected by both molecular and culture-based methods.

^c^Level 3 certainty was considered where there were mild pathological findings, possibly causing death, and the presence of microorganisms that can either represent true pathogens or colonizing/contaminants; mixed infections.

**Supplementary Table 2: Level of certainty for immediate cause of death attribution by the panel.**

|  |  | **Immediate Cause of Death Category** |  | **Level of Certainty** | | |
| --- | --- | --- | --- | --- | --- | --- |
|  |  |  | **N** | **Level 1^a^ (Certain)** | **Level 2^b^ (Probable)** | **Level 3^c^ (Uncertain)** |
| Communicable (Infections) | Meningitis | Hospital-acquired | 2 (1.6%) | 1 (50.0%) | 1 (50.0%) | 0 (0.0%) |
|  |  | Community-acquired^d^ | 4 (3.1%) | 4 (100.0%) | 0 (0.0%) | 0 (0.0%) |
|  | Pneumonia | Hospital-acquired | 11 (8.7%) | 10 (90.9%) | 1 (9.1%) | 0 (0.0%) |
|  |  | Community-acquired^e^ | 31 (24.4%) | 24 (77.4%) | 6 (19.4%) | 1 (3.2%) |
|  | Sepsis | Hospital-acquired | 17 (13.4%) | 12 (70.6%) | 4 (23.5%) | 1 (5.9%) |
|  |  | Community-acquired | 8 (6.3%) | 5 (62.5%) | 2 (25.0%) | 1 (12.5%) |
|  | Other Infections | Bronchiolitis | 2 (1.6%) | 2 (100.0%) | 0 (0.0%) | 0 (0.0%) |
|  |  | Diarrhoea^f^ | 13 (10.2%) | 12 (92.3%) | 1 (7.7%) | 0 (0.0%) |
|  |  | Pulmonary Tuberculosis | 3 (2.4%) | 3 (100.0%) | 0 (0.0%) | 0 (0.0%) |
|  |  | Encephalitis | 2 (1.6%) | 0 (0.0%) | 1 (50.0%) | 1 (50.0%) |
|  |  | Hepatitis | 1 (0.8%) | 1 (100.0%) | 0 (0.0%) | 0 (0.0%) |
|  |  | Intracranial abscess | 1 (0.8%) | 1 (100.0%) | 0 (0.0%) | 0 (0.0%) |
|  |  | Pulmonary mucormycosis | 1 (0.8%) | 1 (100.0%) | 0 (0.0%) | 0 (0.0%) |
| Non-communicable (non-infections) |  | Hyperosmolarity | 1 (0.8%) | 0 (0.0%) | 1 (100.0%) | 0 (0.0%) |
|  |  | Status epilepticus | 1 (0.8%) | 1 (100.0%) | 0 (0.0%) | 0 (0.0%) |
|  |  | Pulmonary embolism | 1 (0.8%) | 1 (100.0%) | 0 (0.0%) | 0 (0.0%) |
|  |  | Other circulatory conditions^g^ | 7 (5.5%) | 4 (57.1%) | 3 (42.9%) | 0 (0.0%) |
|  |  | Aspiration pneumonitis | 2 (1.6%) | 1 (50.0%) | 1 (50.0%) | 0 (0.0%) |
|  |  | Acute Respiratory Distress | 1 (0.8%) | 1 (100.0%) | 0 (0.0%) | 0 (0.0%) |
|  |  | Pulmonary fibrosis | 1 (0.8%) | 0 (0.0%) | 1 (100.0%) | 0 (0.0%) |
|  |  | Hepatic failure | 5 (3.9%) | 5 (100.0%) | 0 (0.0%) | 0 (0.0%) |
|  |  | Gastrointestinal Haemorrhage | 2 (1.6%) | 2 (100.0%) | 0 (0.0%) | 0 (0.0%) |
|  |  | Kidney failure | 1 (0.8%) | 1 (100.0%) | 0 (0.0%) | 0 (0.0%) |
|  |  | Necrotising Enterocolitis | 1 (0.8%) | 0 (0.0%) | 1 (100.0%) | 0 (0.0%) |
|  |  | Tetralogy of Fallot | 1 (0.8%) | 1 (100.0%) | 0 (0.0%) | 0 (0.0%) |
|  |  | Haemorrhage from Respiratory passages | 1 (0.8%) | 1 (100.0%) | 0 (0.0%) | 0 (0.0%) |
|  |  | Sudden Infant Death Syndrome | 1 (0.8%) | 0 (0.0%) | 1 (100.0%) | 0 (0.0%) |
|  |  | Asphyxiation | 1 (0.8%) | 1 (100.0%) | 0 (0.0%) | 0 (0.0%) |
|  |  | Poisoning | 2 (1.6%) | 2 (100.0%) | 0 (0.0%) | 0 (0.0%) |
|  |  | Drowning | 1 (0.8%) | 1 (100.0%) | 0 (0.0%) | 0 (0.0%) |
|  |  | Complications during surgery | 1 (0.8%) | 0 (0.0%) | 0 (0.0%) | 1 (100.0%) |
|  |  | **Total** | **127** | **98 (77.2%)** | **24 (18.9%)** | **5 (3.9%)** |

^a^Level 1 certainty was considered where there were severe pathological findings likely to be the cause of death and the presence of microorganisms that represent true pathogens and/or consistent detection of microorganisms in multiple samples.

^b^Level 2 certainty was considered where there were pathological findings of moderate intensity, probably causing death and the presence of microorganisms that can either represent true pathogens or colonizing/contaminants detected by both molecular and culture-based methods.

^c^Level 3 certainty was considered where there were mild pathological findings, possibly causing death, and the presence of microorganisms that can either represent true pathogens or colonizing/contaminants; mixed infections.

^d^One case of nosocomial *E. coli* meningitis also had nosocomial *P. aeruginosa* pneumonia had co-immediate cause of death

^e^One case of pneumonia also had HIV lymphoid interstitial pneumonitis as a co-immediate cause of death. Another case of nosocomial *S. aureus* pneumonia also had nosocomial disseminated HSV infection as a co-immediate cause of death.

^f^One case of Rotavirus gastroenteritis also had pneumonia as a co-immediate cause of death

^g^One case of congestive heart failure also had congenital hypoplasia and dysplasia of the lung as a co-immediate cause of death.
